# Cardiovascular toxicities after anthracycline and VEGF-targeted therapies in adolescent and young adult cancer survivors

**DOI:** 10.1186/s40959-023-00181-2

**Published:** 2023-07-07

**Authors:** Jeannette R. Wong-Siegel, Robert J. Hayashi, Randi Foraker, Joshua D. Mitchell

**Affiliations:** 1 Division of Pediatric Cardiology, Washington University School of Medicine, St. Louis Children’s Hospital, St. Louis, MO USA; 2 Division of Pediatric Hematology/Oncology, Washington University School of Medicine, St. Louis Children’s Hospital, St. Louis, MO USA; 3 Institute for Informatics, Washington University School of Medicine, St. Louis, MO USA; 4 Cardio-Oncology Center of Excellence, Division of Cardiology, Washington University in St. Louis, 660 S. Euclid Ave, CB 8086, St. Louis, MO 63110 USA

**Keywords:** Cancer therapies, Cancer survivors, Cardiotoxicity

## Abstract

**Background:**

Cancer survival rates have been steadily improving in the adolescent and young adult (AYA) population, but survivors are at increased risk for cardiovascular disease (CVD). The cardiotoxic effects of anthracycline therapy have been well studied. However, the cardiovascular toxicity associated with newer therapies, such as the vascular endothelial growth factor (VEGF) inhibitors, is less well understood.

**Objective:**

This retrospective study of AYA cancer survivors sought to gain insight into their burden of cardiovascular toxicities (CT) following initiation of anthracycline and/or VEGF inhibitor therapy.

**Methods:**

Data were extracted from electronic medical records over a fourteen-year period at a single institution. Cox proportional hazards regression modeling was used to examine risk factors for CT within each treatment group. Cumulative incidence was calculated with death as a competing risk.

**Results:**

Of the 1,165 AYA cancer survivors examined, 32%, 22%, and 34% of patients treated with anthracycline, VEGF inhibitor, or both, developed CT. Hypertension was the most common outcome reported. Males were at increased risk for CT following anthracycline therapy (HR: 1.34, 95% CI 1.04–1.73). The cumulative incidence of CT was highest in patients who received both anthracycline and VEGF inhibitor (50% at ten years of follow up).

**Conclusions:**

CT was common among AYA cancer survivors who received anthracycline and/or VEGF inhibitor therapy. Male sex was an independent risk factor for CT following anthracycline treatment. Further screening and surveillance are warranted to continue understanding the burden of CVD following VEGF inhibitor therapy.

**Supplementary Information:**

The online version contains supplementary material available at 10.1186/s40959-023-00181-2.

## Introduction

Patients diagnosed with cancer between the ages of 15–39 years, the adolescent and young adult (AYA) population, are an understudied group given their historically poor participation in clinical trials [1]. It is further recognized that the incidence of different types of cancer, as well as the biology of site-specific cancers in the AYA population differs from those of children and of older adults [2, 3].

Recent data from the Surveillance, Epidemiology, and End Results (SEER) program noted improved cancer survival rates in the AYA population, with almost 80% surviving more than 5 years after their cancer diagnosis [4]. Despite advances in cancer therapeutics that have contributed to the improving survival rates, survivors remain at increased risk of morbidity and mortality from non-cancer causes, attributed to prior therapies received. Cardiovascular disease (CVD) has emerged as a leading cause of nonrelapse-related mortality among cancer survivors, with more recent reports suggesting that the AYA population are at significant risk [5–8]. Cardiovascular toxicity (CT), including but not limited to hypertension, arrhythmias, and cardiomyopathy, have been associated with a variety of chemotherapeutic agents, with anthracycline therapy demonstrating the most clear association [9].

The cardiotoxic effects of anthracycline are at least partially mediated by topoisomerase-II $\beta$ in cardiomyocytes, resulting in DNA double-strand breaks, defective mitochondrial biogenesis, and reactive oxygen species formation [10]. Over the long-term, these cardiotoxic effects can lead to left ventricular dysfunction and, in the most severe cases, congestive heart failure. High cumulative doses remain the best predictor of eventual cardiac dysfunction, [9] and CT can take several decades before obvious symptomatic changes develop.

Newer, more targeted therapies have emerged for the treatment of cancer, specifically vascular endothelial growth factor (VEGF) inhibitors. VEGF is secreted by tumors and plays a critical role in angiogenesis via the VEGF signaling pathway [11]. VEGF inhibitors have demonstrated promising anti-tumor activity given their ability to target the tumor itself and avoid more systemic toxicity to the patient. Given the role of VEGF in the survival and proliferation of endothelial and vascular smooth muscle cells, adverse cardiovascular effects have been noted acutely with the use of VEGF inhibitors, including hypertension, thrombosis, and cardiomyopathy [12]. However, to date there have been no reports evaluating CT following completion of treatment.

As the cancer incidence rate continues to increase in the AYA population, there is a need to continue to improve or maintain disease-control rates while limiting long-term side effects. Furthermore, given that known CVD risk factors, such as tobacco use, diabetes, and hyperlipidemia, are more prevalent in the AYA than the pediatric population, it is vital to understand the interplay between the patient’s own cardiovascular (CV) risk factors, their cancer therapy, and their risk for future CV disease.

Therefore, we conducted a retrospective study using clinical data obtained from the electronic medical record (EMR) to gain insight into the burden of CTs in an AYA cancer population who received VEGF therapy, comparing these patients to patients exposed to anthracycline therapy, an agent known for its long-term toxicity to the heart.

## Methods

### Study cohort

All AYA patients were identified who were prescribed at least one dose of anthracyclines and/or VEGF inhibitor therapy at Washington University Medical Center in St Louis (including St. Louis Children’s Hospital and Barnes-Jewish Hospital) between January 1, 2004 and June 1, 2018. Patients were required to be between 15 years of age and 39 years of age at time of therapy initiation. Demographics, cancer diagnosis, comorbidities, and outcomes were extracted from the inpatient and outpatient EMRs and the institutional cancer registry. The Washington University in St. Louis Institutional Review Board approved this study.

### Demographic, baseline comorbidities

Demographic information was ascertained for each patient, including sex, race, age, and length of follow-up. Baseline comorbidities before first date of therapy were assessed including CV risk factors (diabetes, hyperlipidemia, and hypertension) and pre-existent CVD (heart failure, coronary disease/angina, and cerebrovascular disease, including stroke and transient ischemic attack) through International Classification of Disease, Ninth Edition (ICD-9) and Tenth Edition (ICD-10) (Supplemental Table 1).

### Therapies of interest

Anthracycline and VEGF inhibitor therapies were queried in the EMR using generic and brand names (Supplemental Tables 2 and 3). Duration of therapy was based on first and last therapy encounters in the EMR.

### Identification of malignancies

Patients were matched to the institutional cancer registry to obtain malignancy diagnoses, tumor site and morphology, and date of cancer diagnosis. Malignancies were then classified according to the International Classification of Childhood Cancer, third edition (ICCC-3) based on the International Classification of Diseases for Oncology, third edition (ICD-O-3) (https://seer.cancer.gov/iccc/iccc3.html).

### Cardiovascular toxicities

CTs were defined at the earliest date of new diagnosis during an inpatient admission or outpatient encounter following initiation of therapy. Events of interest included incident hypertension, coronary artery disease, myocardial infarction, cardiomegaly, cardiomyopathy/heart failure, conduction abnormalities and cerebrovascular events. Events were extracted using ICD-9 and ICD-10 codes (Supplemental Table 4) [13].

### Statistical analysis

Analyses comparing categorical baseline demographic factors and comorbidities by therapies received were calculated using a χ^2^ test when adequate sample sizes were available. Approximate normality for continuous variables was determined graphically by reviewing the histograms and Q-Q plots for each variable’s distribution and statistically using the Shapiro-Wilks test. The Kruskal–Wallis one-way ANOVA was used to compare continuous variables where the normality assumption was not met. Follow-up began on the first date of therapy and continued until the earliest of the following endpoints: date of initial event, death, or at last recorded medical encounter. Chi-squared analysis also assessed the difference in vital status at end of follow-up among the three therapies.

Multivariable Cox proportional hazard regression modeling, adjusted for age at first treatment and sex, evaluated the time-to-event for each CT as well as all-cause mortality among the three treatment groups. Death was included as a competing risk for cardiotoxicity analyses using Fine and Gray’s method. Patients with a diagnosis consistent with a given CT prior to cancer diagnosis were excluded from the assessment of that diagnosis. Additional Cox proportional hazards regression modeling with follow-up as the time scale examined risk factors for development of CT within each therapy modality. Models were adjusted for sex, age at first treatment, and baseline comorbidities, including previous CV diagnoses, diabetes, hyperlipidemia, and hypertension. Cumulative incidences for each CT were also calculated by treatment received, with death as a competing risk. Analyses were again conducted using Fine and Gray’s cumulative incidence method. All analyses were conducted using SAS software (version 9.4; SAS Institute, Cary, NC). Two-sided *p*-values < 0.05 were considered statistically significant.

## Results

In total, 1,306 AYA patients were treated with anthracycline and/or VEGF inhibitor. One hundred and nineteen patients were excluded from the analysis as there was no primary tumor information available from the tumor registry database. An additional 22 patients were excluded due to a benign tumor diagnosis, and 1,165 patients were subsequently included in the analysis (Table 1). Distribution of patients by sex and race were similar across all treatment modalities. Median follow-up for each therapy group was 1.5 years, 0.6 years, and 1.1 years for patients treated with anthracycline only, VEGF inhibitor only, and both, respectively. A small proportion of patients were noted to have pre-existing comorbidities with 8.9% with hypertension, 3.1% diabetes, 4.4% with dyslipidemia, and 1.9% with a pre-existing diagnosis of CVD (Table 1). Patients who were treated with VEGF inhibitor only had the highest prevalence of hypertension prior to initiation of treatment. Only 1% of patients treated with anthracycline only were noted to have cardiomyopathy/heart failure prior to therapy. More patients were alive at the end of the follow-up who received anthracycline only compared to patients treated with VEGF inhibitor in combination or VEGF inhibitor alone. In an age and sex adjusted Cox regression model using the anthracycline only group for reference, there was an increased risk for all-cause mortality in the VEGF alone (aHR 1.80, 95% CI 1.53–2.10) and combined VEGF and anthracycline groups (aHR 2.52, 95% CI 2.16–2.94).

**Table 1:** Demographics of 1,165 oncology patients aged 15–39 years by treatment modality

	**Anthracycline only**		**VEGF inhibitor only**		**Anthracycline and VEGF inhibitor (*N* = 74)**		
	**(*N* = 891)**		**(*N* = 200)**				
	N	(%)	N	(%)	N	(%)	*p*
Sex, n (%)							0.33
Male	443	(50)	88	(44)	35	(47)	
Female	448	(50)	112	(56)	39	(53)	
Race, n (%)							0.16
White	716	(80)	163	(82)	64	(86)	
Black	142	(16)	25	(13)	7	(9)	
Other	33	(4)	11	(6)	3	(4)	
Unknown	0	(0)	1	(0)	0	(0)	
Age at first treatment (y; median, IQR)	29	(23–35)					** < 0.001**
15–19, n (%)	115	(13)	12	(6)	10	(13)	
20–24, n (%)	174	(20)	19	(10)	10	(14)	
25–29, n (%)	184	(21)	34	(17)	13	(18)	
30–34, n (%)	188	(21)	61	(31)	20	(27)	
35–39, n (%)	230	(26)	74	(37)	21	(28)	
Treatment duration, mo. (median, IQR)							
Anthracycline	2	(0.0 – 5.0)	-		2	(0.0 – 7.0)	0.34
VEGF inhibitor	-		3	(0.0 – 9.0)	2	(0.0 – 5.0)	**0.04**
Primary cancer diagnosis, n (%)							** < 0.001**
Brain and spinal	12	(1)	51	(26)	2	(3)	
Leukemia	303	(34)	1	(1)	19	(26)	
Lymphoma	305	(34)	2	(0)	0	(0)	
Sarcoma	122	(14)	31	(16)	26	(35)	
Epithelial/melanoma	134	(15)	104	(52)	10	(14)	
Other solid/unspecified tumors^a^	15	(2)	11	(6)	17	(23)	
Comorbidities prior to treatment, n (%)							
Hypertension	71	(8)	28	(14)	4	(5)	**0.01**
Diabetes	22	(2)	10	(5)	4	(5)	0.09
Dyslipidemia	36	(4)	11	(6)	4	(5)	0.60
Tobacco smoking use, n (%)							0.17
Current	57	(6)	12	(6)	5	(7)	
Former	69	(8)	34	(17)	10	(14)	
Never	765	(86)	154	(77)	59	(80)	
Cardiovascular, n (%)							
Peripheral arterial disease	5	(1)	1	(1)	1	(1)	0.69
Coronary disease	1	(0)	0	(0)	0	(0)	*
Cardiomyopathy/heart failure	10	(1)	1	(0)	0	(0)	*
Cerebrovascular disease	1	(0)	0	(0)	2	(3)	*
Charlson comorbidity index (median, IQR)	0	(0 – 1)	1	(0 – 1)	0	(0 – 1)	** < 0.001**
Vital status, n (%)							** < 0.001**
Alive at last follow-up	613	(69)	87	(43)	16	(22)	
Deceased	278	(31)	113	(57)	58	(78)	

Categorical comparisons were made using chi-squared test. Continuous variables were not normally distributed and were compared using Kruskal–Wallis

^*^Unable to make accurate statistical comparison due to small sample sizes

^a^Includes breast, gastrointestinal, reproductive, and pulmonary malignancies, as well as any malignancies not further specified

There were 290 CT events of interest after initiation of therapy occurring in 26%, 19%, and 30% of patients treated with anthracycline, VEGF inhibitor, or both, respectively (Table 2). The most common outcome for all three treatment groups was incident hypertension, followed by conduction abnormalities. In the anthracycline only group, 1.4 percent of patients subsequently developed cardiomyopathy/heart failure with no incident heart failure noted in the VEGF inhibitor or combined therapy groups during follow-up. In age and sex adjusted Cox proportional hazard models, there was no difference in time-to-event for any of the CTs among the three groups adjusted for the competing risk of death (Table 2).

**Table 2:** Frequency of cardiovascular toxicities by treatment received

	**Anthracycline only**		**VEGF inhibitor only**			**Anthracycline and VEGF inhibitor (*N* = 74)**			
	**(*N* = 891)**		**(*N* = 200)**						
	**N**	**(%)**	**N**	**(%)**	**aHR (95% CI)**	**N**	**(%)**	**aHR (95% CI)**	p*
Any Cardiovascular Toxicity	230	(25.8)	38	(19.0)	0.72 (0.51–1.01)	22	(29.7)	1.06 (0.70–1.61)	0.15
Hypertension	172	(21.0)	30	(17.4)	0.84 (0.57–1.24)	21	(30.0)	1.32 (0.85–2.05)	0.27
Cardiomyopathy/heart failure	12	(1.4)	0	(0)	-	0	(0)	-	*
Cardiomegaly	3	(0.3)	1	(0.5)	-	0	(0)	-	*
Pericardial disease	21	(2.4)	2	(1.0)	-	0	(0)	-	*
Myocardial infarction	4	(0.5)	0	(0)	-	0	(0)	-	*
Coronary artery disease	1	(0.1)	0	(0)	-	0	(0)	-	*
Conduction abnormality/dysrhythmia	47	(5.3)	5	(2.5)	0.45 (0.18–1.13)	2	(2.7)	0.48 (0.12–1.95)	0.15
Cerebrovascular event	3	(0.3)	1	(0.5)	-	0	(0)	-	*
Valvular degeneration	5	(0.6)	1	(0.5)	0.72 (0.09–5.88)	2	(2.8)	4.47 (0.82–24.43)	0.17
Peripheral vascular disease	7	(0.8)	0	(0)	-	0	(0)	-	*

Groups were compared using Cox proportional hazard models adjusted for age and sex to evaluate time until onset of the cardiovascular toxicity. The anthracyline only group was used for the reference. Study participants with a baseline diagnosis of the cardiovasular toxicity were removed from the given analysis. E.g. the 30 patients in the VEGF group with hypertension are new onset hypertension after excluding the 28 patients with baseline hypertension

*CI *Confidence interval

^*^No statistical comparison was made due to infrequent events

Risk factors for development of CT were assessed within each treatment group (Table 3). In the anthracycline only group, male sex was an independent risk factor for developing CT (aHR = 1.40, 95% CI = 1.08–1.81). Non-white race increased the risk for CT in the combined therapy group (aHR = 4.04, 95% CI = 1.35–12.12).

**Table 3:** Risk for any cardiovascular toxicity by therapy received

	**Anthracycline only**					**VEGF inhibitor only**					**Anthracycline + VEGF inhibitor**				
	**Unadjusted**			**Adjusted**		**Unadjusted**			**Adjusted**		**Unadjusted**			**Adjusted**	
	**N**	**HR**	**95% CI**	**HR**	**95% CI**	**N**	**HR**	**95% CI**	**HR**	**95% CI**	**N**	**HR**	**95% CI**	**HR**	**95% CI**
Age at first therapy^a^		1.01	(0.99–1.03)	1.02	(1.00–1.04)		1.00	(0.95–1.05)	1.00	(0.95–1.05)		1.01	(0.95–1.07)	1.02	(0.96–1.09)
Gender															
Female	105	ref.		ref.		20	ref.		ref.		9	ref.		ref.	
Male	131	1.34	(1.04–1.73)	**1.4**	**(1.08–1.81)**	20	1.19	(0.64–2.20)	1.18	(0.63–2.20)	13	1.66	(0.71–3.91)	2.35	(0.93–5.92)
Race															
White	190	ref.		ref.		34	ref.		ref.		17	ref.		ref.	
Non-white	46	1.04	(0.75–1.44)	1.04	(0.75–1.43)	6	0.95	(0.40–2.27)	0.97	(0.41–2.33)	5	2.6	(0.95–7.11)	**4.04**	**(1.35–12.12)**
Comorbidities prior to therapy^b^															
Hypertension	15	1.09	(0.65–1.84)	1.06	(0.62–1.82)	0	-		-		0	-		-	
Diabetes	6	1.44	(0.64–3.24)	1.42	(0.63–3.21)	0	-		-		0	-		-	
Dyslipidemia	8	0.81	(0.40–1.64)	0.75	(0.37–1.54)	2	1.27	(0.30–5.28)	1.27	(0.30–5.36)	2	2.77	(0.62–12.33)	3.54	(0.77–16.35)

Adjusted for age at first therapy, gender, race and comorbidities diagnosed prior to initiation of therapy

^a^Hazard ratios calculated using age at first therapy as a continuous variable per one year increment

^b^Reference group: patients without respective comorbidity prior to treatment

Among patients in the anthracycline only group, we also assessed risk factors for the development of any major cardiac event, such as myocardial infarction, coronary artery disease, heart failure, and cerebrovascular accident (Table 4). When adjusting for age at first therapy, gender, and race, pre-existing hypertension was significantly associated with an increased risk for a major cardiac event (aHR = 4.54, 95% CI = 1.41–14.63). This analysis was not performed in the other therapy groups due to insufficient sample size.

**Table 4:** Risk for any major cardiac event^a^ among patients who received anthracycline

	**Anthracycline**				
	**Unadjusted**			**Adjusted**	
	**N**	**HR**	**95% CI**	**HR**	**95% CI**
Age at first therapy^b^		1.04	(0.97 – 1.12)	1.04	(0.97 – 1.12)
Gender					
Female	6	ref.		ref.	
Male	12	2.15	(0.81 – 5.72)	2.41	(0.89 – 6.50)
Race					
White	14	ref.		ref.	
Non-white	4	1.19	(0.39 – 3.62)	1.14	(0.37 – 3.50)
Comorbidities prior to therapy^c^					
Hypertension	4	**4.29**	**(1.40 – 13.10)**	**4.54**	**(1.41 – 14.63)**
Diabetes	0	–		–	
Dyslipidemia	0	–		–	

Adjusted for age at first therapy, gender, race and comorbidities diagnosed prior to initiation of therapy

^a^Any major cardiac event defined as myocardial infarction, coronary artery disease, heart failure, or cerebrovascular accident

^b^Hazard ratios calculated using age at first therapy as a continuous variable per one year increment

^c^Reference group: patients without respective comorbidity prior to treatment

**Fig. 1:**
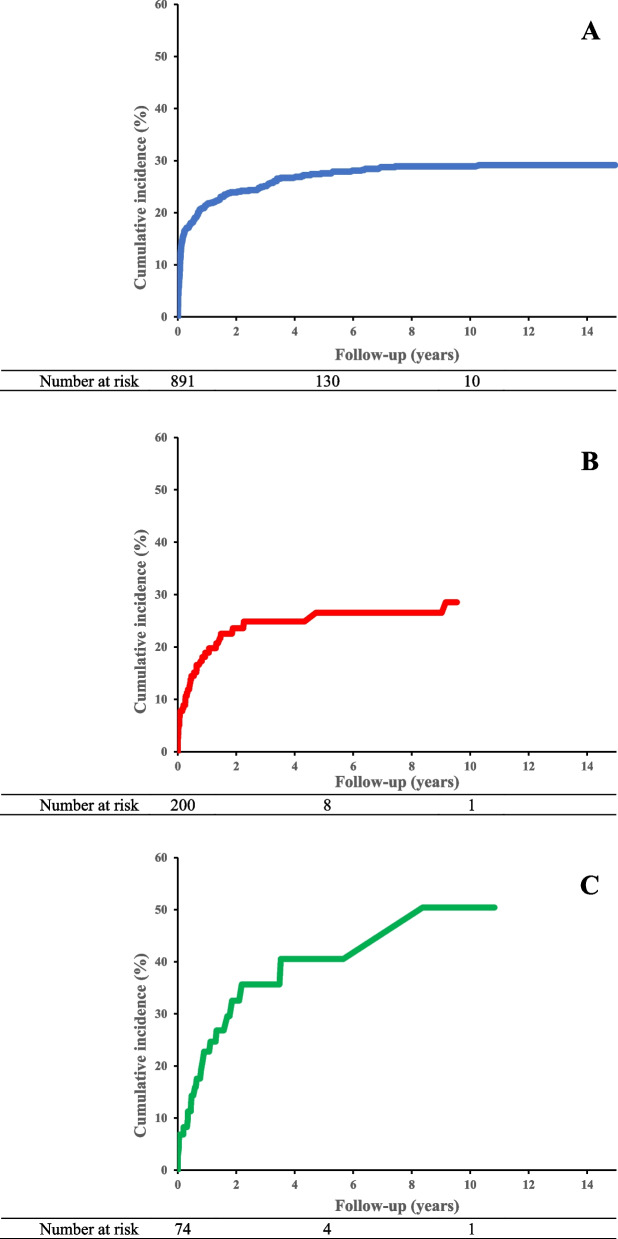
Cumulative incidence of any cardiovascular toxicity by therapy received: (**A**) Anthracycline only (**B**) VEGF inhibitor only (**C**) Anthracycline and VEGF inhibitor

The cumulative incidence of CT, with death as a competing risk, was then evaluated for each treatment group (Fig. 1). Patients who received both anthracycline and VEGF inhibitor were noted to have a cumulative incidence of 50% at 10 years of follow up, compared to approximately 29% in patients who received anthracycline or VEGF inhibitor only. Furthermore, as noted in Table 1, mortality significantly differed among the treatment modalities, with 78% of patients who received combined therapy deceased by the end of follow-up compared to 31% and 57% among patients treated with anthracycline or VEGF inhibitor only, respectively (χ^2^ = 97.1, *p* < 0.001).

## Discussion

Increased long-term risk for CT outcomes among cancer survivors who received anthracycline has been previously reported, but few studies have elucidated the CV impacts of the newer cancer therapies, such as those agents that target the VEGF signaling pathway. Furthermore, there are limited data on the CV outcomes among the AYA cancer survivor population in general due to low clinical trial participation. To our knowledge, this is the first study to describe the CTs observed in an AYA cancer survivor cohort who received VEGF inhibitor therapy. Over a third of patients developed CT, and, by comparison, the frequency of CT was similar to those who received anthracycline only. Higher cumulative incidence of CT was observed among patients who received both therapies.

Male sex was the only independent risk factor for CT in patients who received anthracyclines. Female sex has previously been reported to be a risk factor for anthracycline-associated CT among pediatric cancer survivors [14–19]. However, the role of sex as a risk factor among adults is less conclusive with only a few studies noting increased risk of CT among males [20, 21]. Interestingly, adult animal models of anthracycline-associated CT have consistently demonstrated significant cardioprotection among female rodents compared to males, which may be contributed to differences in mitochondrial function and the detrimental role of male sex hormones [22].

Non-white race was also a significant risk factor for CT among patients who received both anthracycline and VEGF inhibitor therapy. However, given small numbers and heterogeneity of this group, further investigation is needed to understand this possible association.

Hypertension was the most frequently observed CT in all three treatment groups. While hypertension is a well-established potential side effect of VEGF inhibitor therapy, studies have reported varying levels of incidence. A comprehensive meta-analysis of VEGF inhibitor studies reported an average incidence rate of 7.4% for severe hypertension among cancer patients [23]. Increased oxidative stress secondary to anthracycline therapy has also been described to contribute to the development of hypertension, which may further increase subsequent risk for heart failure/cardiomyopathy [24]. While the interaction of anthracycline and VEGF inhibitor therapy may exacerbate the risk for hypertension overall, further analyses to elucidate this hypothesis was limited given small numbers in our study.

Hypertension is a notable CT, as the incidence of hypertension alone increases the risk of future cardiomyopathy by a factor of 17 in survivors of childhood cancer treated with anthracyclines [25]. Indeed, in our own study, baseline hypertension increased the risk of future major cardiac event by a factor of 4. Clinicians should be alert to the importance of hypertension screening and treatment in this at-risk population to reduce their risk of CV morbidity and mortality.

While VEGF inhibitors pose a risk for CT, they remain an essential part of treatment for many cancers, improving overall survival or progression-free survival [26]. The incidence of VEGF inhibitor-associated acute cardiotoxicity appears to be very rare with at most 3% of patients in a randomized trial experiencing high grade congestive heart failure as defined by the NCI common terminology criteria for adverse events [27]. However, in one randomized trial involving use of anthracycline and VEGF inhibitor patients with hepatocellular carcinoma, 19% of patients experienced some form of CT [28]. Here, we report an increased cumulative incidence of CT, as well as a significantly higher proportion of deceased patients, among those who received both therapies. These patients were likely at higher risk for mortality based on the cancer type and stage, and cancer therapy selection should continue to prioritize overall survival. Managing cardiovascular risk and toxicity in parallel, though, can hopefully reduce the need for cancer therapy cessation and improve long-term morbidity and mortality [29].

### Limitations

There are several limitations to this study. We did not have a control group of AYA patients without cancer to calculate the expected proportion of CT, although our incidence rates are significantly higher than the prevalence in the general population [30]. We could not account for unmeasured confounders, such as lifestyle factors (diet, exercise). Due to home administration of anthracyclines in some patients and limitations of the EMR, we were unable to confidently ascertain each patient’s cumulative anthracycline dose. The proportion of patients experiencing CT after initiation of therapy could be underestimated if patients were seen and treated outside our healthcare system. Some CTs could be transiently related to initiation of therapy, though these short-term complications may have relevant long-term implications. While we cannot statistically compare the rate of CT across treatment groups due to varying cancer diagnoses and other population differences, it is helpful to understand each group in isolation to target further investigation and management strategies.

## Conclusions

This study represents the first to describe CT among AYA cancer survivors after receiving VEGF inhibitor therapy, comparing the observed incidence to patients receiving anthracycline or both. The incidence of CT was high in all three groups, reaching 40% cumulative incidence by 4 years in the combination therapy group. While VEGF inhibitors deliver clear benefits in cancer treatment, these patients appear to warrant close CV monitoring as we begin to understand the long-term impacts of therapy on cancer survivorship. Further investigations are needed to better define the full scope of this clinical problem.

## Supplementary Information

**Additional file 1: Supplemental Table 1.** Definition of pre-existing comorbidities and cardiovascular disease. **Supplemental Table 2. **Anthracycline therapies included in analysis. **Supplemental Table 3.** VEGF inhibitor therapies included in analysis. **Supplemental Table 4. **Definition of cardiovascular toxicities.

## Data Availability

Not applicable.

## Abbreviations

AYA: Adolescent and young adult
CI: Confidence interval
CT: Cardiovascular toxicity
CV: Cardiovascular
CVD: Cardiovascular disease
EMR: Electronic medical record
HR: Hazard ratio
SEER: Surveillance, Epidemiology, and End Results
VEGF: Vascular endothelial growth factor
