# Supplementary material for: Cardiovascular toxicities after anthracycline and VEGF-targeted therapies in adolescent and young adult cancer survivors
Source: Cardiooncology. 2023 Jul 7;9:30. doi: 10.1186/s40959-023-00181-2 (PMC10327375; doi:10.1186/s40959-023-00181-2)
Supplement: Supplementary file 1 — Additional file 1: Supplemental Table 1. Definition of pre-existing comorbidities and cardiovascular disease. Supplemental Table 2. Anthracycline therapies included in analysis. Supplemental Table 3. VEGF inhibitor therapies included in analysis. Supplemental Table 4. Definition of cardiovascular toxicities. [file 40959_2023_181_MOESM1_ESM.docx]

| **Supplemental Table 1:** Definition of pre-existing comorbidities and cardiovascular disease | | |
| --- | --- | --- |
| **Comorbidities** | **ICD-9** | **ICD-10** |
| HTN | 401X, 402X, 403X, 404X 405X 642X | I10X, I11X, I12X, I13X, I15X, O16X |
| Diabetes | 250.x | E10.x, E11.x, E12.x, E13.x |
| Dyslipidemia | 272.0, 272.1, 272.2, 272.4, 272.8, 272.9 | E78.0x, E78.1, E78.2, E78.4, E78.5 |
| **Cardiovascular disease** |  |  |
| Cardiomyopathy/Heart Failure | 402.01, 402.11, 402.91, 404.01, 404.03, 404.11, 404.13, 404.91, 404.93, 425.5, 425.8, 425.9, 428.x | I11.0, I13.0, I13.2, I25.5,  I42.0, I42.5, I42.7-9, I43.x, I50.x |
| Acute myocardial infarction | 410.x | I21.x-I22.x |
| Other acute/subacute ischemic heart disease | 411.1, 411.81, 411.89 | I24.0, I24.8-9 |
| Cerebrovascular accidents | 433.x, 434.x | I63.x |
| Transient ischemic attacks | 435.x | G45.x |

| **Supplemental Table 2.** Anthracycline therapies included in analysis. | |
| --- | --- |
| **Generic** | **Trade Name** |
| Daunorubicin | Daunoxome |
|  | Cerubidine |
| Doxorubicin | Rubex |
|  | Adriamycin |
|  | Doxil |
|  | Lipodox |
|  | Vyxeos |
| Epirubicin | Ellence |
|  | Pharmorubicin |
| Idarubicin | Idamycin |
| Mitoxantrone | Novantrone |
| Valrubicin | Valstar |

| **Supplemental Table 3.** VEGF inhibitor therapies included in analysis. | |
| --- | --- |
| **VEGF inhibitor** | **Trade Name** |
| Aflibercept | Zaltrap |
| Axitinib | Inlyta |
| Bevacizumab | Avastin |
| Cabozantinib | Cabometyx |
|  | Cometriq |
| Lenvatinib | Lenvima |
| Nintedanib | Ofev |
|  | Vargatef |
| Pazopanib | Votrient |
| Ramucirumab | Cyramza |
| Regorafenib | Stivarga |
| Sorafenib | Nexavar |
| Sunitinib | Sutent |
| Vandetanib | Caprelsa |

| **Supplemental Table 4.** Definition of cardiovascular toxicities. | | | |
| --- | --- | --- | --- |
|  | **ICD-9** | **ICD-10** |  |
| Hypertension | 401.x, 402.x, 403.x, 404.x, 405.x, 642.x | I10.x, I11.x, I12.x, I13.x, I15.x, O16.x |  |
| Cardiomyopathy/Heart Failure | 402.01, 402.11, 402.91, 404.01, 404.03, 404.11, 404.13, 404.91, 404.93, 425.5, 425.8, 425.9, 428.x | I11.0, I13.0, I13.2, I25.5,  I42.0, I42.5, I42.7-9, I43.x, I50.x |  |
| Cardiomegaly | 429.3 | I51.7 |  |
| Pericardial disease | 420.x, 423.x | I30.x, I31.x |  |
| Myocardial infarction  Acute myocardial infarction | 410.x | I21.x-I22.x |  |
| Other acute and subacute forms of ischemic heart disease | 411.1, 411.81, 411.89 | I24.0, I24.8-9 |  |
| Coronary atherosclerosis | 414.0 | I25.0, I25.1 |  |
| Conduction abnormalities |  |  |  |
| Conduction disorders | 426.x | I44.x – I48.x |  |
| Cardiac dysrhythmias | 427.x | I49.x |  |
| Cerebrovascular event |  |  |  |
| Cerebrovascular accidents | 433.x, 434.x | I63.x |  |
| Transient ischemic attacks | 435.x | G45.x |  |
| Valvular degeneration | 424.x | I34.x, I35.x, I36.x, I37.x |  |
| Peripheral vascular disease | 440.x, 441.x, 443.x, 444.x, 445.x, 447.x, 557.x, 250.7x | I70.x, I71.x, I73.x, I74.4, I77.1, I79.x, K55.1, K55.8, K55.9, Z95.9, E08.5x, E09.5x, E10.5x, E11.5x, E13.5x |  |
